# Interventions for prevention and treatment of trastuzumab-induced cardiotoxicity: an umbrella review of systematic reviews and meta-analyses

**DOI:** 10.3389/fphar.2024.1479983

**Published:** 2024-12-05

**Authors:** Yunfang Wang, Jianguo Xu, Yafei Xie, Dan Zhou, Mingyue Guo, Yu Qin, Kang Yi, Jinhui Tian, Tao You

**Affiliations:** ^1^ Department of Endocrinology, Gansu Provincial Hospital, Lanzhou, China; ^2^ Evidence-Based Medicine Center, School of Basic Medical Sciences, Lanzhou University, Lanzhou, China; ^3^ Department of Thoracic Surgery, West China Hospital, Sichuan University, Chengdu, China; ^4^ Department of Gastroenterology and Hepatology, Sichuan University-University of Oxford Huaxi Joint Centre for Gastrointestinal Cancer, West China Hospital, Sichuan University, Chengdu, China; ^5^ Department of Obstetrics, Xiangya Hospital Central South University, Changsha, China; ^6^ Department of Rehabilitation Sciences, The Hong Kong Polytechnic University, Kowloon, Hong Kong SAR, China; ^7^ Department of Cardiovascular Surgery, Gansu Provincial Hospital, Lanzhou, China; ^8^ Gansu International Scientific and Technological Cooperation Base of Diagnosis and Treatment of Congenital Heart Disease, Lanzhou, China; ^9^ Key Laboratory of Evidence-based Medicine of Gansu Province, Lanzhou University, Lanzhou, China

**Keywords:** trastuzumab, cardiotoxicity, cardioprotective strategies, meta-analysis, umbrella review

## Abstract

**Background:**

Trastuzumab therapy for HER2-positive cancers is associated with cardiotoxicity. This umbrella review synthesizes evidence from systematic reviews and meta-analyses on cardioprotective interventions during trastuzumab treatment.

**Methods:**

A comprehensive search was conducted in PubMed, Embase, Cochrane Library, and Web of Science. Systematic reviews and meta-analyses examining cardioprotective interventions in patients receiving trastuzumab were included. The methodological quality was assessed using the AMSTAR-2 tool. Data on cardiac events, treatment interruptions, left ventricular ejection fraction (LVEF) changes, and exercise interventions were synthesized.

**Results:**

Ten systematic reviews met the inclusion criteria. Statins demonstrated the strongest cardioprotective effect (RR = 0.47, 95% CI: 0.26–0.84), potentially preventing more than half of cardiac events during trastuzumab therapy, followed by beta-blockers (RR = 0.61, 95% CI: 0.39–0.93). Beta-blockers and ACEIs effectively reduced treatment interruptions, enabling approximately 40% more patients to maintain treatment continuity (RR = 0.63, 95% CI: 0.47–0.86). Among non-pharmacological interventions, structured exercise programs showed significant benefits in preserving cardiac function, demonstrating meaningful improvements in resting LVEF (WMD = −3.27%, 95% CI: −5.86 to −0.68).

**Discussion:**

This review demonstrates that cardioprotective interventions, particularly statins and beta-blockers, significantly reduce the risk of cardiac complications during trastuzumab therapy. The positive impact on cardiac events and treatment interruptions suggests these interventions may enhance overall treatment efficacy by allowing more patients to complete their prescribed course.

**Conclusion:**

Evidence strongly supports the systematic implementation of cardioprotective strategies in clinical practice, particularly statins and beta-blockers, as part of routine care protocols for patients receiving trastuzumab therapy. These interventions demonstrate significant potential in preventing cardiac complications and maintaining treatment continuity. Further research should focus on optimizing personalized approaches and evaluating long-term outcomes.

## 1 Introduction

As a chronic disease that seriously affects human health, the incidence of malignant tumors is increasing year by year worldwide, imposing a heavy burden on countries around the world (Sleeman et al., 2019). The main feature of malignant tumors is infinite division, and growth factors and their receptors play a key role in the growth and differentiation of cells. Abnormal expression of human epidermal growth factor receptor 2 (Her-2) is seen in many common malignancies such as breast cancer, ovarian cancer, gastric cancer, colorectal cancer, etc. Her-2-positive tumor cells have higher growth-stimulating viability, which in turn causes rapid tumor growth and spread (Hung and Lau, 1999). Trastuzumab is a Her-2 monoclonal antibody that inhibits the growth of Her-2-positive tumor cells by inhibiting the interaction between growth factors and tumor cells and has significant clinical efficacy (Kulhari et al., 2015). Trastuzumab can be used as monotherapy or in combination with chemotherapeutic agents such as anthracyclines, paclitaxel and 5-fluorouracil to improve the efficacy (Costa et al., 2010; Hurvitz et al., 2018). Drug-induced cardiotoxicity is one of the common complications of trastuzumab use, manifested by clinical signs of reduced left ventricular ejection fraction (LVEF), arrhythmias and even heart failure (Gabani et al., 2021). Trastuzumab causes cardiotoxicity by various mechanisms, including damage to cardiomyocytes by triggering antibody-dependent cytotoxicity on the one hand (Zambelli et al., 2011), and damage to cardiomyocytes by inhibiting normal cardiomyocyte survival signaling pathways due to excessive inhibition of Her-2 on the other (Xia et al., 2002). Cardiotoxicity affects the prognosis of patients, and in particular, delayed cardiotoxicity has a dramatic impact on the quality of survival and even survival rates of patients. Epidemiological surveillance found that cardiovascular events were the main cause of death in patients over 66 years old with breast cancer, accounting for 15.9% of the deaths (Patnaik et al., 2011). This significant cardiovascular mortality emphasizes the urgent need for effective cardioprotective strategies, particularly in older patients who may have pre-existing cardiovascular risk factors, to optimize both cancer treatment outcomes and overall survival. Therefore, the detection and intervention of trastuzumab-induced cardiotoxicity without compromising the efficacy of chemotherapy is a hot research topic in the field related to trastuzumab, which is important to improve patient prognosis. Despite advances in targeted therapy, the management of trastuzumab-induced cardiotoxicity remains challenging, with significant variations in prevention strategies and monitoring approaches across clinical practices. The optimal timing and choice of cardioprotective interventions, especially in high-risk populations, remain unclear.

Unlike irreversible cardiotoxicity due to anthracycline mechanism, cardiotoxicity due to trastuzumab mechanism is reversible because there are few ultrastructural changes in cardiomyocytes (Gabani et al., 2021). This reversibility presents a crucial therapeutic window where appropriate interventions could prevent permanent cardiac damage while maintaining the benefits of HER2-targeted therapy. Therefore, the management of trastuzumab-induced cardiotoxicity focuses on monitoring, supplemented by intervention. Monitoring of cardiotoxicity includes imaging monitoring (ECG, echocardiography) and biomarker monitoring (CK-MB, cTnI, cTnT), and related models are converging to personalized medicine construction (Dokmanovic and Wu, 2015). Recent studies on pharmacological interventions have focused on restoring myocardial function and slowing down ventricular remodeling with the cardioprotective agents dexrazoxane, angiotensin-converting enzyme inhibitor (ACEI), angiotensin receptor blocker (ARB), beta-blocker (BB), statins, etc (Geuna et al., 2020; Calvillo-Arguelles et al., 2019). Some studies have shown that exercise also has a role in the improvement of cardiotoxicity (Hojan et al., 2020). The decision to discontinue or not to discontinue the drug based on the monitoring results of cardiotoxicity, optimize the combination of chemotherapeutic drugs, control the dose and change the mode of administration, and supplement with cardioprotective drugs to improve cardiac function have become the mainstream measures to prevent and treat trastuzumab-induced cardiotoxicity. While various cardioprotective interventions have shown promise, their relative effectiveness and optimal implementation timing remain debated. Current evidence presents inconsistent findings across different prevention strategies, with varying levels of methodological quality and potential biases affecting the interpretation of results. The heterogeneity in study designs and outcomes assessment methods further complicates the translation of research findings into clinical practice.

The prevention and treatment of trastuzumab-induced cardiotoxicity has been extensively explored in original studies and systematic reviews, but the evidence remains fragmented and sometimes contradictory. This umbrella review addresses these limitations by systematically synthesizing and evaluating the quality of available evidence across all intervention types. By providing a comprehensive analysis of cardioprotective strategies, this review aims to offer healthcare providers and researchers clear, evidence-based guidance for optimizing cardiac monitoring and management in patients receiving trastuzumab. Through integration of findings from multiple high-quality studies, we seek to establish a more robust framework for clinical decision-making in the treatment of HER2-positive cancers.

## 2 Methods

### 2.1 Inclusion criteria

#### 2.1.1 Types of participants

Patients with any type, progressive stage, or severity of cancer, receiving trastuzumab either as monotherapy or in combination with other HER2-targeted therapies and chemotherapy drugs, regardless of age, gender, or race. This approach was taken to reflect real-world clinical practice where combination therapies are common.

#### 2.1.2 Interventions and comparisons

The control group was a chemotherapy regimen with or without placebo on trastuzumab alone or in combination with other chemotherapy drugs. The treatment group was the chemotherapy regimen used in the control group plus cardioprotective agents or exercise. For pharmacological interventions, there were no restrictions on dosage form, dose, duration of treatment, route of administration, or manufacturer. Exercise interventions included structured aerobic and/or resistance training programs following established guidelines, excluding non-conventional exercise forms such as yoga or tai chi.

#### 2.1.3 Outcomes

We determined the preventive and therapeutic effects of the intervention on trastuzumab-induced cardiotoxicity by comparing the changes in LVEF, changes in cardiac function, and the incidence of cardiac events in the control and intervention groups before and after treatment. The primary outcome indicators were the incidence of cardiotoxic events, changes in echocardiographic monitoring parameters, and Her-2 treatment interruptions. Cardiotoxic events refer to all kinds of heart diseases caused by drugs during chemotherapy, particularly cardiac dysfunction defined as a decline in LVEF to below 50% or an absolute decrease of ≥10 percentage points from baseline, symptomatic heart failure, or significant arrhythmias (Barish et al., 2019). HER2 treatment interruptions refer to temporary or permanent discontinuation of trastuzumab therapy due to cardiac dysfunction, defined as either a decline in LVEF to below 50% or an absolute decrease of ≥10 percentage points from baseline, or clinical signs and symptoms of heart failure. These interruptions are initiated based on standardized cardiac monitoring protocols and may be temporary with resumption of therapy once cardiac function recovers, or permanent if cardiac dysfunction persists or is severe.

#### 2.1.4 Types of studies

Only systematic reviews (with or without meta-analysis) were included, and their included studies should be randomized controlled trials (RCTs) or contain observational studies. A systematic review is defined as a review that included each of the following items: a research question, search strategy and sources, inclusion and exclusion criteria, screening methods, quality assessment of included studies, and analysis and integration of data.

### 2.2 Exclusion criteria

Reviews that used theoretical studies, qualitative data, or texts and opinions as their major source of evidence, such as descriptive systematic reviews and systematic reviews of animal studies, were excluded. Other types of reviews including mapping reviews, scoping reviews, narrative reviews, and reviews without systematic methodology were excluded. Duplicate literature, conference papers, letters, errata, protocol, etc. were similarly excluded. Outcome indicators that did not include information on effectiveness or safety, such as effectiveness rates and adverse events, were excluded, too. Literature that needed to be excluded due to outcome indicators included: outcome indicators that needed to be analyzed separately for descriptive reasons such as large heterogeneity; combined data that could not be extracted for mean differences, ratio ratios, and relative risks; outcome indicators with apparently incorrect outcome data or inconsistent images; and outcome indicators with inconsistent descriptions of results and conclusions.

### 2.3 Search strategy

Our search experts developed a comprehensive search strategy with a combination of subject terms and free words, using Boolean logic operators, with no language restrictions. The search results were obtained from the following databases: Pubmed, Embase, Cochrane Library and Web of Science. Our search formula in PubMed was as follows: (“Cardiotoxicity” [Mesh] or “Cardiotoxicity” or Cardiotoxicities or “Cardiac Toxicity” or “Cardiac Toxicities”) and (“Meta-Analysis” [Publication Type] OR “Meta-Analysis as Topic” [Mesh] OR “meta analysis” [Title/Abstract] OR “meta analyses” [Title/Abstract] OR meta-analysis [Title/Abstract] OR meta-analyses [Title/Abstract] OR “systematic review” [Title/Abstract] OR “systematic reviews” [Title/Abstract] OR metaanalysis [Title/Abstract] OR metaanalyses [Title/Abstract]). For the other three databases we constructed search formulas for searching in the same way. Reference lists of eligible reviews and meta-analyses were searched for additional citations. All databases were searched from their inceptions to 4 April 2022, and the entire search process was repeated on 20 May 2024, for the last time (Elliott et al., 2017).

### 2.4 Study selection

The retrieved records were all imported into EndNote X9 and all duplicate files were eliminated using the automatic check function. The first stage was a blind independent screening by two reviewers in the title and abstract, followed by cross-checking. Any conflicts were resolved through on-site discussion with a third reviewer. All reviews included in the first phase were screened in full-text. If they meet the above inclusion criteria, they would be included in the review.

### 2.5 Quality assessment

The online AMSTAR 2 (A MeaSurement Tool to Assessing systematic Reviews) assessment scale is used to assess methodological quality and to provide an overall rating for the included systematic reviews (Shea et al., 2017). The AMSTAR-2 assessment scale contains 16 items related to systematic review, seven of which are considered critical. The item assessment criteria are divided into “Yes,” “Partial Yes” and “No.” There are four categories according to “Overall Confidence”: high, medium, low and very low. All systematic reviews meeting our inclusion criteria were included regardless of their AMSTAR-2 ratings, but these quality assessments were considered when interpreting the strength of evidence and discussed in our findings.

### 2.6 Data collection

The data extraction form was co-designed by two researchers and consisted of two parts: the systematic review data extraction form and the original study data extraction form, which were modified and refined by pre-testing. The above two researchers independently extracted the data and then cross-checked them, and a third researcher intervened to reach a consensus in case of disagreement. Information extracted from systematic reviews included: title, year, author, journal, search time and sources, participants, interventions, comparisons, quality assessment tools, sample size, and outcome indicators. For each systematic review, we extracted both the total sample size of all included studies and the outcome-specific sample sizes, as the number of participants varied across different outcome analyses within the same review. Original study literature refers to literature from reference lists of included systematic reviews or database searches, of which patients were on trastuzumab alone or in combination, extracting information including: title, year, author, journal, participants, interventions, comparisons, sample size and outcome indicators.

### 2.7 Data synthesis

The unit of analysis for this umbrella review consisted of systematic reviews and meta-analyses meeting our inclusion criteria. For quantitative synthesis, we extracted original data from primary studies included in these reviews and conducted new meta-analyses, ensuring each study contributed data only once to our analyses (Xie et al., 2024). Results were systematically presented in tabular format, incorporating numerical data from the overall effect assessments and meta-analyses of the included reviews. For each outcome, we reported the number of primary studies, total participants, and heterogeneity measures from the included reviews. We acknowledged and accounted for the overlap of primary studies across multiple systematic reviews to avoid duplicate representation of data. Qualitative synthesis employed narrative analysis to summarize and interpret textual data from the reviews. This approach was also used for topic summaries and quality assessments of the literature.

For quantitative data, we conducted a new meta-analysis using Stata 14.2 software. We extracted original data from the included reviews and their primary studies where available. Heterogeneity was assessed using Q and I^2^ statistics. Based on the degree of heterogeneity, we selected random-effects model to calculate pooled effect sizes and their 95% confidence intervals (CI). When data were unclear or incomplete in systematic reviews, we consulted the original publications. Studies with missing outcome data were excluded from meta-analyses but included in the qualitative synthesis. Given that our meta-analyses mainly focused on objective cardiac outcomes with standardized measurements, missing data were minimal and primarily related to incomplete reporting rather than loss to follow-up. Given the limited number of studies for each outcome, formal assessment of publication bias was not conducted. We performed comprehensive subgroup analyses to evaluate the effectiveness of interventions across different clinical contexts and to assess the robustness of our findings.

## 3 Results

### 3.1 Literature screening results and basic characteristics of included studies

**FIGURE 1:**
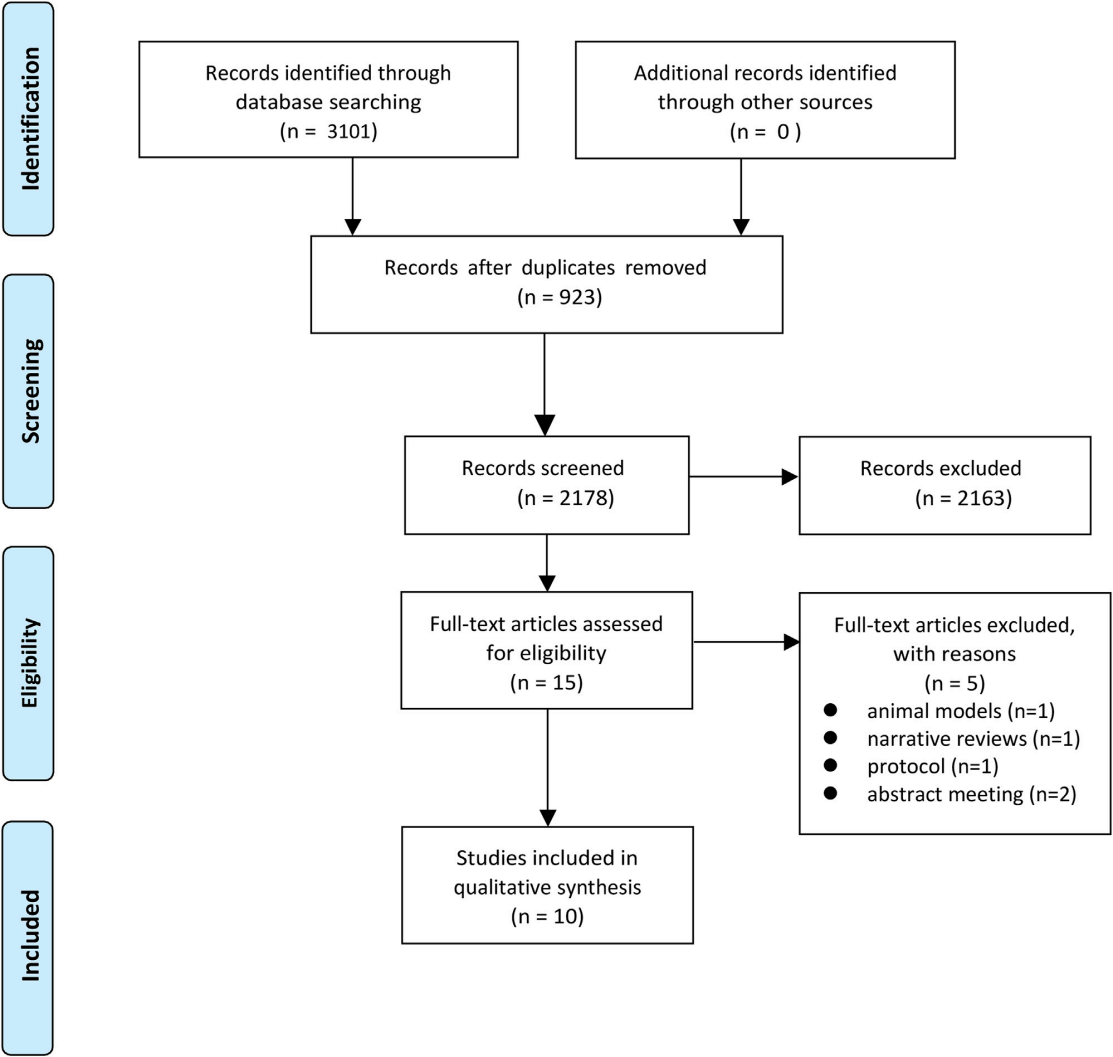
Flow diagram of literature screening and selection process.

Our comprehensive search across multiple databases yielded a total of 3,101 records. After removing 923 duplicates, 2,178 records were screened based on titles and abstracts. Of these, 2,163 were excluded as they did not meet the inclusion criteria. The remaining 15 full-text articles were assessed for eligibility, resulting in the further exclusion of five articles. Ultimately, 10 systematic reviews and meta-analyses (Kalam and Marwick, 2013; Gujral et al., 2018; Dong et al., 2020; Elghazawy et al., 2020; Antunes et al., 2019; Brown et al., 2021; Fang et al., 2021; Lewinter et al., 2021; Obasi et al., 2021; Li et al., 2021) were included in our study. The literature screening process is illustrated in Figure 1.

The 10 included studies were published between 2013 and 2021, encompassing a range of cardioprotective interventions. Seven studies (Gujral et al., 2018; Dong et al., 2020; Elghazawy et al., 2020; Brown et al., 2021; Fang et al., 2021; Lewinter et al., 2021; Li et al., 2021) focused on combinations of cardioprotective drugs (such as ACEI, ARB, and BB), one study investigated statins (Obasi et al., 2021), one examined dexrazoxane along with other drugs (Kalam and Marwick, 2013), and one explored exercise interventions (Antunes et al., 2019). The study populations primarily consisted of patients receiving HER2-targeted therapy, predominantly trastuzumab for breast cancer. The included primary studies were a mix of RCTs and observational studies, with sample sizes ranging from 154 to 2,105 participants across the systematic reviews. The main outcome measures across these studies included changes in LVEF, incidence of cardiotoxic events, diagnosis of heart failure, and interruptions in HER2-targeted therapy. Most studies utilized the Cochrane Risk of Bias Tool for quality assessment. Table 1 provides a detailed overview of each study’s characteristics, including the specific interventions, search strategies, number of included studies, sample sizes, and key findings with their respective effect sizes and confidence intervals.

**TABLE 1:** Characteristics of included systematic reviews and meta-analyses.

Studies	Therapeutic drugs	Retrieval time	Search database	Risk of bias assessment tools	N of studies	Total sample size	Outcome	Effect size	95% CI	Outcome-specific sample size
Kalam and Marwick (2013)	Dexrazoxane, Statins, ACEI, BB	N/A	Embase, Medline, EBSCO HOST	N/A	14	2,105	events (Overall effect)	RR = 0.31	[0.25,0.39]	2,105
							events (Dexrazoxane)	RR = 0.35	[0.27,0.45]	1,162
							events (BB)	RR = 0.31	[0.16,0.63]	458
							events (Statins)	RR = 0.31	[0.13,0.77]	241
							events (ACEI)	RR = 0.11	[0.04,0.29]	244
Gujral et al. (2018)	ACEI, ARB, BB	2017.01	Embase, PubMed Cochrane library	N/A	8	1,052	mean change in LVEF (ACEI/ARB)	MD = −4.74	[−12.56,3.08]	324
							mean change in LVEF (BB)	MD = −3.28	[−6.06,−0.51]	252
							heart failure diagnosis (ACEI)	OR = 0.24	[0.03,1.73]	530
							heart failure diagnosis (BB)	OR = 0.33	[0.14,0.8]	622
Dong et al. (2020)	ACEI, ARB	2019.11	Embase, PubMed Cochrane library	RoB Tool	5	702	events (ACEI/ARB)	OR = 0.91	[0.62,1.34]	581
							risk of hypotension (ACEI/ARB)	OR = 2.72	[0.69,10.73]	581
							mean change in LVEF (ACEI/ARB)	MD = 4.08	[0.8,7.35]	702
Elghazawy et al. (2020)	ACEI, ARB, BB	2019.02	PubMed, Scopus, Cochrane library, Web of Science	RoB Tool	22	N/A	mean change in LVEF (ACEI/ARB)	SMD = −2.58	[−3.94,−1.22]	872
							mean change in LVEF (ACEI/ARB/BB)	SMD = −2.36	[−3.23,−1.49]	1,758
							mean change in LVEF (BB)	SMD = −1.45	[−2.27,−0.64]	1,308
							heart failure diagnosis (BB)	OR = 0.12	[0.03,0.45]	562
							mean change in ESV (ACEI/ARB/BB)	SMD = −3.22	[−6.55,0.12]	380
							mean change in EDV (ACEI/ARB/BB)	SMD = 1.71	[−1.93,5.35]	380
							mean change in ESD (ACEI/ARB/BB)	SMD = −1.2	[−2.89,0.49]	456
							mean change in EDD (ACEI/ARB/BB)	SMD = −1.11	[−1.88,−0.35]	648
Antunes et al. (2019)	Aerobic exercise, Resistance exercise	2020.05	Medline, Scopus, Cochrane library, Web of Science, ClinicalTrials.gov, ICTRP	RoB Tool	4	154	resting left ventricular ejection fraction (exercise)	MD = 2.09	[−0.17,4.34]	147
							longitudinal strain (exercise)	MD = −0.31	[−2.07,1.46]	71
							E/A ratio (exercise)	MD = 0.09	[−0.08,0.25]	135
Brown et al. (2021)	ACEI, ARB, BB	2021.01	Embase, Scopus, Medline Cochrane Library ICTRP	RoB Tool	5	952	events (Overall effect)	RR = 0.9	[0.63,1.29]	952
							events (BB)	RR = 0.52	[0.1,2.66]	371
							events (ACEI/ARB)	RR = 0.94	[0.55,1.62]	581
							HER2 therapy treatment interruptions (Overall effect)	RR = 0.57	[0.43,0.77]	740
							HER2 therapy treatment interruptions (BB)	RR = 0.54	[0.36,0.83]	369
							HER2 therapy treatment interruptions (ACEI/ARB)	RR = 0.55	[0.29,1.04]	371
							mean change in LVEF (Overall effect)	MD = −1.50	[−2.16,−0.83]	876
							mean change in LVEF (ACEI/ARB)	MD = −1.74	[−2.18,−1.30]	371
							mean change in LVEF (BB)	MD = −1.49	[−2.82,−0.16]	502
Fang et al. (2021)	ACEI, ARB	2019.07	PubMed, Embase, Cochrane Library	RoB Tool	9	1,095	events (ACEI/ARB)	RR = 0.63	[0.3,1.31]	799
							risk of hypotension (ACEI/ARB)	RR = 3.94	[1.42,10.9]	647
							mean change in LVEF (ACEI/ARB)	MD = 4.24	[1.53,6.95]	1,095
Lewinter et al. (2021)	ACEI, ARB, BB	2021.03	PubMed, Embase, Cochrane Library	RoB Tool	9	1,362	mean change in LVEF (ACEI/ARB)	MD = 1.5	[−0.6,3.7]	681
							mean change in LVEF (BB)	MD = 2.4	[0.3,4.5]	941
							mean change in LVEF (patients treated with anthracyclines)	MD = 1.9	[−0.5,4.2]	708
							mean change in LVEF (patients treated with trastuzumab)	MD = 2.3	[0.4.6]	914
Obasi et al. (2021)	Statins	N/A	PubMed, Embase, Web of Science, ClinicalTrials.gov, Cochrane Library	RoB Tool NOS	6	930	events (Observational)	RR = 0.462	[0.275,0.776]	N/A
							events (RCT)	RR = 0.495	[0.169,1.451]	N/A
							mean change in LVEF (Observational)	WMD = 6.137	[2.753,9.52]	N/A
							mean change in LVEF (RCT)	WMD = 6.248	[0.82,11.676]	N/A
Li et al. (2021)	ACEI, ARB, BB	2021.02	Embase, PubMed, Cochrane Library	RoB Tool	8	N/A	events (ACEI)	OR = 0.48	[0.057,2.3]	N/A
							events (BB)	OR = 0.49	[0.057,2.3]	N/A
							HER2 therapy treatment interruptions (ACEI)	OR = 0.45	[0.12,1.3]	N/A
							HER2 therapy treatment interruptions (ARB)	OR = 0.87	[0.15,4.8]	N/A
							HER2 therapy treatment interruptions (BB)	OR = 0.41	[0.11,1.2]	N/A

Abbreviation: ACEI, Angiotensin-Converting Enzyme Inhibitor; ARB, angiotensin receptor blocker; BB, Beta-Blocker; LVEF, left ventricular ejection fraction; RCT, randomized controlled trial; ICTRP, international clinical trials registry platform; NOS, Newcastle-Ottawa Scale; N/A, not applicable.

### 3.2 Quality evaluation of included studies

**FIGURE 2:**
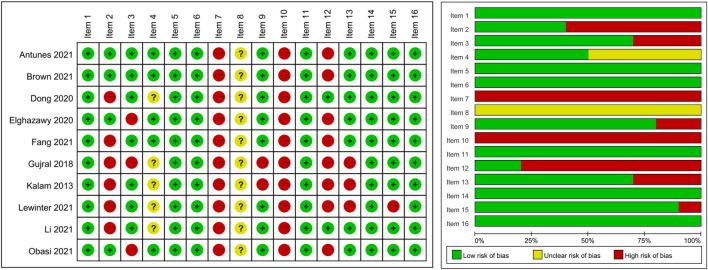
Methodological quality assessment of included systematic reviews using the AMSTAR 2 tool. Note: Item 1: PICO components in research question and inclusion criteria; Item 2: Protocol registered before review conduct; Item 3: Explanation for selection of study designs; Item 4: Comprehensive literature search strategy; Item 5: Duplicate study selection; Item 6: Duplicate data extraction; Item 7: List and justify excluded studies; Item 8: Detailed description of included studies; Item 9: Appropriate technique for assessing risk of bias in individual studies; Item 10: Report on sources of funding for included studies; Item 11: Appropriate methods for statistical combination of results in meta-analysis; Item 12: Assessment of potential impact of risk of bias on meta-analysis results; Item 13: Account for risk of bias in individual studies when interpreting results; Item 14: Explanation and discussion of heterogeneity; Item 15: Adequate investigation of publication bias and discussion of its likely impact; Item 16: Report potential sources of conflict of interest.

The methodological quality of the included systematic reviews was assessed using the AMSTAR 2 tool, with results presented in Figure 2. Overall, the quality of the included studies varied across the 16 AMSTAR 2 items. The methodological quality varied considerably across the included studies. Most studies performed well in defining research questions with PICO components (Item 1). However, significant limitations were observed in several critical areas: protocol registration (Item 2) was inadequate in most studies, comprehensive literature searches (Item 4) and descriptions of included studies (Item 8) showed inconsistent quality across reviews, and none of the studies provided a complete list of excluded studies with justifications (Item 7). The assessment of publication bias (Item 15) was adequately addressed in about half of the reviews. Risk of bias assessment (Item 13) and heterogeneity discussion (Item 14) were appropriately conducted in most studies, though there was room for improvement in some reviews.

### 3.3 Synthesis of outcome indicators and key findings

Our analysis synthesized data from original studies included in the systematic reviews and meta-analyses that met our inclusion criteria. We categorized our findings into four key areas: cardiac events, HER2 therapy treatment interruptions, changes in LVEF, and exercise intervention outcomes. These categories encompass the primary endpoints in cardioprotection during cancer treatment, offering insights into both the incidence of adverse cardiac effects and the efficacy of preventive strategies. The following sections present a comprehensive synthesis of our findings in each area, based on our new meta-analyses of the original study data.

#### 3.3.1 Cardiac events

**FIGURE 3:**
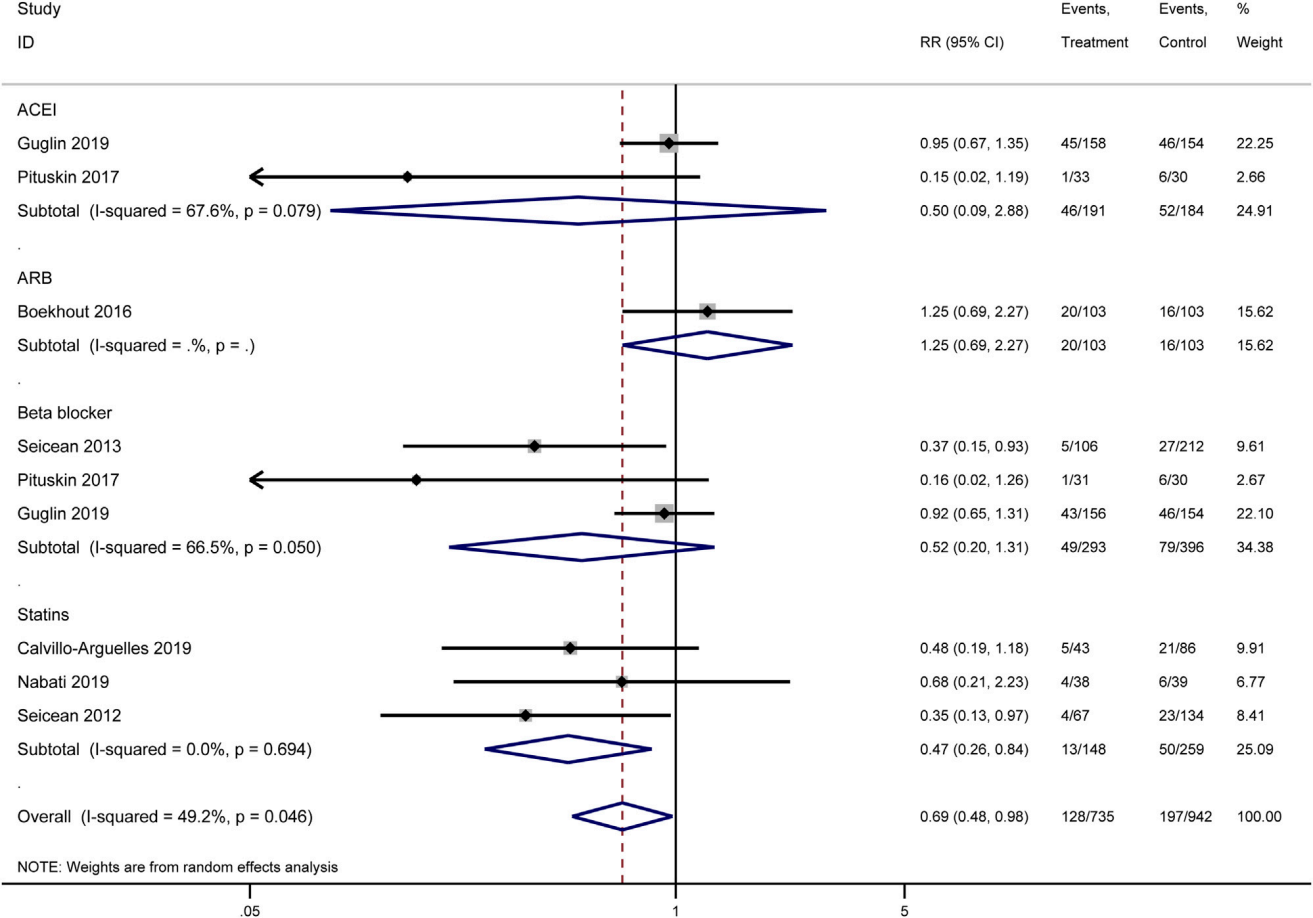
Forest plot of the effect of cardioprotective interventions on cardiac events.

Our meta-analysis synthesized the results of multiple original studies (Calvillo-Arguelles et al., 2019; Guglin et al., 2019; Pituskin et al., 2017; Boekhout et al., 2016; Seicean et al., 2013; Nabati et al., 2019; Seicean et al., 2012) examining the impact of various cardioprotective interventions on cardiac events (Figure 3). The overall effect of pharmacological interventions indicated a potential reduction in cardiac event risk (RR = 0.69, 95% CI: 0.48–0.98, I^2^ = 49.2%, p = 0.046). Among the analyzed drug categories, statins demonstrated the most pronounced cardioprotective effect. Pooled analysis results showed that statins potentially reduced the risk of cardiac events significantly (RR = 0.47, 95% CI: 0.26–0.84), with low heterogeneity across studies (I^2^ = 0.0%, p = 0.694). This consistent effect suggests that statins may be particularly effective in reducing cardiac events in patients receiving HER2-targeted therapies. Beta-blockers showed a trend towards cardioprotection, though not reaching statistical significance (RR = 0.52, 95% CI: 0.20–1.31, I^2^ = 66.5%, p = 0.050). The effect of ACEIs was inconclusive due to high heterogeneity between studies (RR = 0.50, 95% CI: 0.09–2.88, I^2^ = 67.6%, p = 0.079), indicating variability in the reported outcomes across different original studies. For ARBs, only one study was included, which did not show a clear protective effect (RR = 1.25, 95% CI: 0.69–2.27).

Heterogeneity was observed in some subgroups, particularly for ACEIs and beta-blockers, as reflected in the I^2^ values and p-values for heterogeneity tests. This variability suggests that the effectiveness of these interventions may depend on various clinical and methodological factors not uniformly accounted for across the included studies.

#### 3.3.2 HER2 therapy treatment interruptions

**FIGURE 4:**
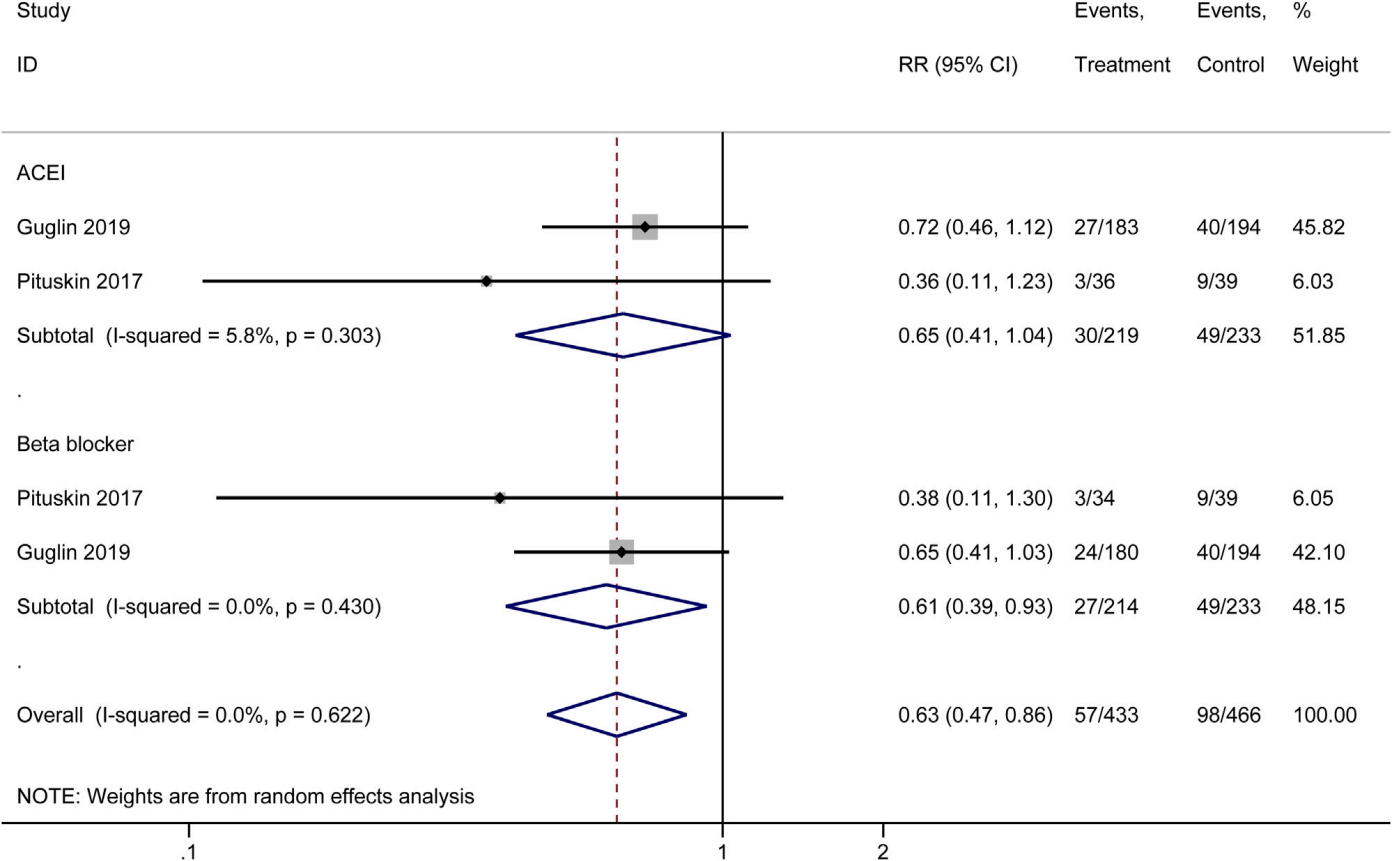
Forest plot of the effect of cardioprotective interventions on HER2 therapy treatment interruptions.

Our meta-analysis synthesized data from multiple original studies examining the impact of cardioprotective interventions on HER2 therapy treatment interruptions (Figure 4). The forest plot presents the results for two main categories of interventions: ACEIs and beta-blockers. For ACEIs, two studies were included (Guglin 2019 (Guglin et al., 2019) and Pituskin 2017 (Pituskin et al., 2017)). The pooled effect estimate showed a trend towards reduced treatment interruptions, although not statistically significant (RR = 0.65, 95% CI: 0.41–1.04). The heterogeneity between these studies was low (I^2^ = 5.8%, p = 0.303), suggesting consistency in their findings. Beta-blockers also demonstrated a potentially beneficial effect in reducing HER2 therapy interruptions. The studies by Pituskin 2017 (Pituskin et al., 2017) and Guglin 2019 (Guglin et al., 2019) yielded a statistically significant result (RR = 0.61, 95% CI: 0.39–0.93). There was no observed heterogeneity between these studies (I^2^ = 0.0%, p = 0.430), indicating concordance in their results.

The overall pooled effect across both ACEI and beta-blocker interventions showed a significant reduction in HER2 therapy treatment interruptions (RR = 0.63, 95% CI: 0.47–0.86). The lack of heterogeneity in the overall analysis (I^2^ = 0.0%, p = 0.622) suggests consistency in the protective effect of these interventions across the included original studies. These findings indicate that both ACEIs and beta-blockers may play a role in reducing the frequency of HER2 therapy treatment interruptions, with beta-blockers showing a statistically significant effect. The overall analysis supports the potential benefit of these cardioprotective interventions in maintaining continuity of HER2-targeted cancer therapies.

#### 3.3.3 Mean change in left ventricular ejection fraction (LVEF)

**FIGURE 5:**
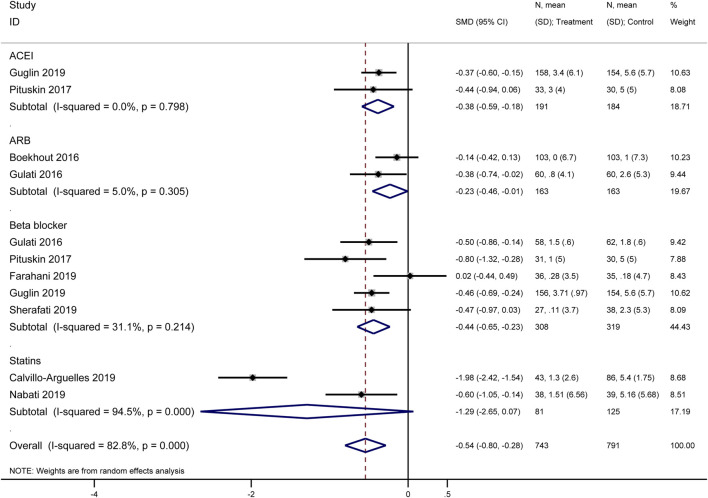
Forest plot of the effect of cardioprotective interventions on mean change in Left Ventricular Ejection Fraction (LVEF).

Our meta-analysis synthesized data from multiple original studies (Guglin et al., 2019; Pituskin et al., 2017; Boekhout et al., 2016; Gulati et al., 2016; Farahani et al., 2019; Sherafati et al., 2019) examining the impact of various cardioprotective interventions on the mean change in LVEF (Figure 5). The forest plot presents results for four categories of interventions: ACEIs, ARBs, beta-blockers, and statins. ACEIs showed a significant protective effect on LVEF (SMD = −0.38, 95% CI: −0.59 to −0.18). The two included studies demonstrated consistency in their findings, with no heterogeneity observed (I^2^ = 0.0%, p = 0.798). ARBs also demonstrated a significant protective effect (SMD = −0.23, 95% CI: −0.46 to −0.01). The analysis included two studies (Boekhout 2016 and Gulati 2016) with low heterogeneity (I^2^ = 5.0%, p = 0.305). Beta-blockers showed the most consistent protective effect among the pharmacological interventions (SMD = −0.44, 95% CI: −0.65 to −0.23). This subgroup analysis included five studies with moderate heterogeneity (I^2^ = 31.1%, p = 0.214). Statins demonstrated the largest effect size (SMD = −1.29, 95% CI: −2.65 to 0.07), although with significant heterogeneity between the two included studies (I^2^ = 94.5%, p = 0.000). This high heterogeneity suggests caution in interpreting these results.

The overall pooled effect across all interventions indicated a significant protective effect on LVEF (SMD = −0.54, 95% CI: −0.80 to −0.28). However, substantial heterogeneity was observed in the overall analysis (I^2^ = 82.8%, p = 0.000), likely due to the variability in effect sizes across different drug classes and individual studies.

These findings suggest that cardioprotective interventions, particularly ACEIs, ARBs, and beta-blockers, may have a beneficial effect on preserving LVEF in patients receiving HER2-targeted therapies. The results for statins, while promising, require further investigation due to the high heterogeneity observed.

#### 3.3.4 Exercise intervention outcomes

Our analysis identified one original study (Hojan et al., 2020) that examined the effects of exercise interventions on cardiac function in patients receiving HER2-targeted therapies. This study reported outcomes for three key cardiac parameters. For resting left ventricular ejection fraction, the exercise intervention showed a significant improvement compared to the control group (WMD = −3.27, 95% CI: −5.86 to −0.68). Regarding longitudinal strain, a trend towards improvement was observed, although not statistically significant (WMD = −0.60, 95% CI: −2.04 to 0.84). The E/A ratio showed no significant difference between the exercise and control groups (WMD = 0.00, 95% CI: −0.30 to 0.30). These results indicate varying effects of exercise interventions on different cardiac parameters in patients undergoing HER2-targeted therapies, with the most notable impact observed on resting left ventricular ejection fraction. However, as this analysis is based on a single study, further research is needed to confirm these findings and explore their generalizability across different patient populations and exercise intervention protocols.

## 4 Discussion

This umbrella review synthesized evidence from 10 systematic reviews and meta-analyses on strategies to prevent and manage cardiotoxicity in patients receiving trastuzumab therapy. Through rigorous methodological quality assessment using AMSTAR-2, we identified varying quality across included reviews, with strengths in PICO formulation and risk of bias assessment, but limitations in protocol registration and documentation of excluded studies. Despite these methodological limitations, our findings corroborate and extend existing literature, providing stronger evidence for the efficacy of cardioprotective medications in mitigating HER2-targeted therapy-associated cardiac risks. Statins, beta-blockers, and ACEIs demonstrated particular potential in reducing cardiac events and minimizing treatment interruptions (Zamorano et al., 2016; Curigliano et al., 2012; Armenian et al., 2017). While based on limited data, our review also offers targeted evidence for exercise interventions in this specific patient population. The significant effects observed on LVEF preservation across various interventions underscore the importance of early cardioprotective strategies. By consolidating high-quality evidence from diverse studies, our review provides a nuanced understanding of the relative effectiveness of different interventions, offering valuable insights for future clinical practice guidelines and research directions to optimize the balance between cancer treatment efficacy and cardiovascular health preservation. Our findings both complement and extend the existing literature in this field. A previous meta-review (Conway et al., 2015) provided valuable insights into the broad spectrum of cancer treatment-induced cardiotoxicity; however, our umbrella review focuses specifically on trastuzumab-related cardiotoxicity, presenting updated evidence through 2024. Additionally, while prior research (Dempsey et al., 2021) explored clinical risk factors and pharmacologic prevention strategies for trastuzumab-induced cardiotoxicity, our analysis offers a more comprehensive quantitative synthesis of intervention effectiveness derived from multiple systematic reviews. Our results regarding the efficacy of beta-blockers and ACE inhibitors align with earlier conclusions (Pun and Neilan, 2016), yet provide more precise effect estimates through our updated meta-analyses. Furthermore, this review expands on previous work by including detailed analyses of exercise interventions and presenting comparative effectiveness data across various cardioprotective strategies.

Cardioprotective interventions demonstrated significant benefits in reducing cardiac events, minimizing treatment interruptions, and preserving LVEF in patients undergoing trastuzumab therapy. Statins emerged as particularly promising, demonstrating the most significant reduction in cardiac events (RR = 0.47, 95% CI: 0.26–0.84) with consistent results across studies. This finding aligns with the known pleiotropic effects of statins, including their anti-inflammatory and antioxidant properties, which may contribute to cardioprotection during cancer treatment (Paris et al., 2020; Wright and Lefer, 2005; Davignon, 2004). While statins demonstrate promising cardioprotective effects, their use requires careful consideration of potential risks and contraindications in cancer patients. Liver function monitoring is particularly important as both statins and chemotherapy can affect hepatic function. The included studies reported generally acceptable safety profiles, with manageable adverse events primarily including mild myalgias and reversible liver enzyme elevations. Notably, no significant interactions between statins and trastuzumab were reported in the analyzed studies. However, careful attention should be paid to potential drug-drug interactions, particularly in patients receiving multiple chemotherapy agents or supportive medications. The timing of statin initiation and dose optimization may also be crucial factors in minimizing adverse effects while maintaining cardioprotective benefits.

Beta-blockers and ACEIs also showed potential benefits, particularly in reducing HER2 therapy treatment interruptions (RR = 0.61, 95% CI: 0.39–0.93 for beta-blockers) and preserving LVEF. Among beta-blockers, cardioselective agents (particularly metoprolol and bisoprolol) were most commonly studied and showed consistent cardioprotective effects. These agents’ selective β1-receptor blockade may offer advantages in cancer patients by minimizing peripheral effects while maintaining cardiac protection. Carvedilol, a non-selective beta-blocker with additional α-blocking and antioxidant properties, demonstrated particular promise in several included studies, possibly due to its multiple mechanisms of action. Regarding ACEIs, both tissue-selective agents (ramipril, perindopril) and non-tissue selective agents (enalapril) demonstrated cardioprotective effects, with tissue-selective ACEIs showing slightly more favorable outcomes in terms of LVEF preservation. The protective effects of these drugs are likely mediated through their ability to modulate neurohormonal activation and cardiac remodeling (Strauss et al., 2023; Manolis et al., 2023). The cardioprotective mechanisms appear to involve reduction of sympathetic overactivation, prevention of adverse cardiac remodeling, and maintenance of optimal hemodynamics during cancer therapy. Notably, timing of initiation emerged as a crucial factor, with preventive administration showing superior outcomes compared to reactive treatment.

While limited to one original study, our review found that exercise interventions significantly improved resting LVEF (WMD = −3.27, 95% CI: −5.86 to −0.68). This finding underscores the potential role of structured physical activity in maintaining cardiac function during cancer treatment, possibly through improved cardiovascular fitness and reduced systemic inflammation.

The implications of these findings are far-reaching for the management of patients receiving trastuzumab therapy. Cardioprotective medications, particularly statins, beta-blockers, and ACEIs, emerge as key considerations for patients at high risk of cardiotoxicity (Avila et al., 2018). These interventions may play a crucial role in maintaining cancer treatment continuity by reducing the need for therapy interruptions (Zamorano et al., 2016; Curigliano et al., 2012). Regular cardiac monitoring, including LVEF assessment, remains crucial. The significant effects of interventions on LVEF preservation suggest that early initiation of cardioprotective strategies may be beneficial. Implementing a structured cardiac surveillance program for all patients undergoing trastuzumab therapy could facilitate timely detection of cardiac dysfunction and guide early interventions, potentially preventing more severe complications (Armenian et al., 2017). Incorporating structured exercise programs into patient care may offer additional cardioprotective benefits. While the evidence is limited, the positive impact of exercise on resting LVEF is promising. Exercise programs should be tailored to individual patient capabilities and closely supervised. Given the variability in outcomes across different interventions, a personalized approach to cardioprotection is warranted (Jones et al., 2019). Developing comprehensive risk assessment tools that consider individual patient factors can guide the selection and intensity of cardioprotective interventions, allowing for more nuanced patient care.

The complex interplay between cancer treatment and cardiovascular health necessitates close collaboration among healthcare professionals. Establishing multidisciplinary cardio-oncology teams can facilitate integrated care, optimize treatment decisions, and ensure comprehensive management of both oncological and cardiovascular aspects. Additionally, the potential for delayed cardiotoxicity emphasizes the importance of long-term cardiac surveillance, even after completing trastuzumab therapy. Establishing protocols for extended follow-up and clear pathways for cardiology referrals may help in the early detection and management of late-onset cardiac complications (Curigliano et al., 2016; Abdel-Qadir et al., 2019). Implementing these evidence-based strategies can help clinicians optimize patient outcomes, balancing effective cancer treatment with cardiovascular health preservation. Further research will be crucial in refining these approaches and evaluating their long-term impact.

Our umbrella review has identified several critical areas warranting further investigation to advance cardioprotection in trastuzumab therapy. Comparative effectiveness studies represent a crucial next step, as direct head-to-head comparisons of interventions are lacking. Such studies would enable clinicians to make evidence-based decisions tailored to individual patient needs (Esfandbod et al., 2021). The optimal timing and duration of cardioprotective interventions remain unclear, necessitating research into whether initiating these strategies prior to, concurrent with, or following trastuzumab therapy yields the best outcomes. Biomarker research holds significant promise in personalizing cardioprotective strategies, potentially improving cost-effectiveness by focusing resources on high-risk patients (Urun et al., 2015). While our review primarily focused on breast cancer patients, further investigation is needed to apply these findings to non-breast cancer populations receiving trastuzumab therapy. Long-term follow-up studies are essential to fully understand the impact of cardioprotective interventions on both immediate effects and long-term cardiovascular outcomes and overall survival. Exploring combination strategies that leverage both pharmacological and non-pharmacological interventions could yield valuable insights into more comprehensive and effective cardioprotection approaches. Addressing these research priorities requires collaborative efforts across multiple disciplines, including large-scale, multicenter trials and real-world studies to generate robust, generalizable evidence. By pursuing these research directions, we can continue to refine our approach to cardioprotection in trastuzumab therapy, ultimately improving the balance between effective cancer treatment and long-term cardiovascular health for patients undergoing HER2-targeted therapies.

Several limitations should be noted in interpreting the findings of this umbrella review. Significant heterogeneity was observed in some analyses, particularly for statins’ effect on LVEF, potentially reflecting differences in study populations, dosing regimens, or concomitant treatments across primary studies. The generalizability of findings is limited, as most included studies focused on breast cancer patients, constraining applicability to other cancer types treated with trastuzumab. Additionally, the reviewed studies primarily reported short to medium-term outcomes, leaving long-term cardiovascular effects and survival impacts of these interventions less clear. Another limitation is that many included systematic reviews did not comprehensively report demographic characteristics such as age, race, and ethnic background of their study populations. This common limitation in evidence synthesis studies restricts our ability to explore these important patient-specific factors as potential sources of heterogeneity in treatment effects. While we included only high-quality systematic reviews, the possibility of publication bias in the primary studies cannot be entirely ruled out. These limitations highlight the need for further research to address these gaps and strengthen the evidence base for cardioprotective strategies in trastuzumab therapy.

## 5 Conclusion

This umbrella review synthesizes high-quality evidence on cardioprotective strategies for patients receiving trastuzumab therapy, offering crucial insights into the efficacy of various interventions. Our findings highlight the potential of pharmacological approaches, particularly statins, beta-blockers, and ACE inhibitors, as well as structured exercise programs, in mitigating trastuzumab-associated cardiotoxicity. While primarily derived from breast cancer studies, these findings may inform cardioprotective strategies across other HER2-positive cancers with appropriate consideration of cancer-specific factors. These results have significant implications for clinical practice, suggesting that a multifaceted approach to cardioprotection may effectively preserve cardiovascular health without compromising cancer treatment efficacy. By providing a comprehensive overview of current knowledge, this review lays the foundation for more personalized and proactive management of cardiovascular risks in patients undergoing HER2-targeted therapies. Implementation of these evidence-based strategies has the potential to reduce cardiac complications, improve treatment adherence, and ultimately enhance both short-term and long-term outcomes for cancer patients.

## Data Availability

The original contributions presented in the study are included in the article/supplementary material, further inquiries can be directed to the corresponding author.
